# Extreme temperature events and their relationship with excess all-cause mortality in Chandigarh, India

**DOI:** 10.1038/s41598-025-32614-6

**Published:** 2026-01-29

**Authors:** Ravindra Khaiwal, Prachi Chauhan, Sanjeev Bhardwaj, Abhishek Kumar, Suman Mor

**Affiliations:** 1 Department of Community Medicine and School of Public Health, Post Graduate Institute of Medical Education and Research (PGIMER), Chandigarh, 160012 India; 2 Department of Environment Studies, Panjab University, Chandigarh, 160014 India

**Keywords:** Extreme temperature, Temperature threshold, Heatwave, All-cause mortality, Correlation, Environmental sciences, Environmental social sciences

## Abstract

Climate change has increased the frequency and intensity of extreme temperature events, adversely impacting human health and mortality. This study examines daily all-cause mortality concerning daily maximum and minimum temperature over a six-year period (2010–2015) in Chandigarh, India. Using an over-dispersed Poisson Generalized Additive Model (GAM), with visibility as a surrogate for other meteorological factors, we found a strong model fit (R^2^ = 0.996; *P* < 0.05). Our results show a significant increase in all-cause mortality during heatwave conditions. A moderately positive association between temperature and mortality was observed in summer (R^2^ 0.014 in May; R^2^ = 0.133 in June; *p* < 0.05), while a negative association was found in winter (r = −0.155 in December; r =  − 0.141 in January; *p* < 0.05). A critical temperature threshold of 33.8 °C (*p* = 0.0007) was identified, above which mortality significantly increases, although the effect size remains modest. Mortality risk was found to be elevated during heatwaves, with similar risk levels observed for both males and females. These findings highlight the need for targeted interventions and adaptation strategies to reduce temperature-related mortality, particularly in vulnerable populations exposed to extreme weather conditions.

**Supplementary Information:**

The online version contains supplementary material available at 10.1038/s41598-025-32614-6.

## Introduction

Climate change has influenced not only the global average climate but also extreme temperatures. These temperature extremes are getting severe, and every year, a large number of deaths are recorded due to these harsh weather conditions^1, 2, 3^. Some extreme weather events that were rare earlier have now become more frequent, like droughts, flash floods, and cyclones^4^, with heatwaves causing a further deficit in soil moisture, crop yield, and affecting food security^5^. These events are expected to become more severe as climate change becomes pronounced^6, 7, 8^, with a study showing that heatwave events would increase across the cities for all periods under SSP 370 and 585 scenarios^9, 10^. Extreme temperature events occur in the form of heat and cold waves. A heatwave is when hot and sometimes humid weather conditions prevail for a prolonged duration. Whereas, during cold waves the temperature remains low for a longer duration, sometimes even due to invasion by a cold air mass^11^.

The average global temperature has increased by about 1.29 °C (2.32 °F) above the 20^th^-century average^12^. Two-thirds of the warming has occurred since 1975, at a rate of roughly 0.15–0.20 °C per decade^13^. It is now possible to confidently attribute some weather events like heatwaves to rising global temperatures^14, 15, 16^. The International Panel for Climate Change (IPCC) Assessment Report^17^ (AR5), which was released in 2015, found that net annual temperatures in India in the 2030s, compared to the 1970s, will increase from 1.7 to 2.2 °C. Extreme temperatures are expected to increase by 1–4 °C, with a maximum increase in coastal regions^13^,[Ravindra, 2025] . By 2100, there can be an increase in the number of warm days and nights by 55% and 70% respectively under the RCP8.5 scenario^18^. Temperature extremes cause weather-related mortality^19^ (Gasparrini et al.^20^).

Extreme weather conditions, such as heatwaves, can disrupt the body’s physiological processes and homeostasis, which is the regulatory mechanism that maintains the core body temperature at around 37 °C. When the air temperature rises above 37 °C, the body starts gaining heat from the air. Approximately 90% of heat dissipation occurs through the skin, and in high relative humidity conditions, evaporation slows down, leading to excessive perspiration and water loss but no cooling effect. This can result in heat-related illnesses like fainting, cramps, and heat exhaustion^21^. In cases of high humidity, the human body can experience heat stress even at temperatures around 37 °C and 38 °C. The heat index measures the perceived temperature by factoring in both air temperature and relative humidity. For instance, if the air temperature is 34 °C with 70% humidity, the heat index would be 47 °C, producing a similar effect as a temperature of 31 °C with 95% humidity^22^.

Cardiovascular and pulmonary-related mortality is more prevalent at low temperatures than at high temperatures^23, 24^. People with pre-existing chronic conditions have a higher risk of dying in heatwaves^25^. Morbidity due to temperature also depends upon the vulnerability of the individual. Economically marginalized and older adults are directly exposed to heatwaves, and their vital signs respond sensitively to temperature increases^26, 27^. Heat-related morbidity varies across age groups, from younger to older individuals^28^, and is influenced by immunocompromised status and socio-economic factors^29, 30^. Seniors and disabled individuals are more susceptible to mortality^31^, and urban residents are more vulnerable compared to those in rural areas^32^.

Recently, the impact of temperature extremes has been felt globally [Bhardwaj et al., 2025]. Heatwave exposure has disproportionately increased in the lowest-income regions globally compared to the highest-income areas over the past four decades^33^. There is considerable evidence from the literature that daily mortality is correlated with current and preceding day ambient temperature^34^. Several studies have been conducted to study the impact of extreme temperatures on excess all-cause mortality. A study in tropical countries like Brazil shows that females are more vulnerable to cold than heat. In contrast, males are more vulnerable to cerebrovascular diseases and ischemic stroke during heat. Mortality is higher on heatwave days, and cardiovascular and respiratory-related mortality is high in females^35^. The mortality during heatwaves shows age and sex-dependent variation. Mortality is 45% higher in females aged 45 and above. Most of the deaths occurred directly due to heat stroke and hyperthermia. Individual acclimatization and susceptibility affect the mortality rate. Community characteristics also play a role in influencing mortality rates. Mortality risk increases with the duration and intensity of heatwaves. High mortality has been seen for heatwaves that occur early in summer^36, 37^.

In the case of cold waves, the mortality rate is higher with cold waves that occur late in the season^38, 21^. Climate change is expected to result in higher heat-related mortality and lower cold-related mortality^39^. In extreme cold conditions, the body loses heat faster than it can produce it. This leads to the utilization of energy stored in the body and causes hypothermia, where body temperature falls below 35 ℃. This also affects the brain and normal cognitive functioning^40^. Excessive temperature extremes affect those who are unable to regulate and maintain their core body temperature due to some pre-existing conditions like chronic disease, excessive use of alcohol and drugs, old age, and infants^21^.

Over the past several years, there has been a systematic increase in the frequency, total duration, and maximum duration of heatwaves in India, severely affecting numerous cities^41^. These extreme heat events have caused significant loss of human life, with about 3000 deaths in 1998, over 2000 in 2002, and more than 2400 in 2015^42, 32^. Heatwaves also cause the death of cattle and wildlife, as well as affect animals in various zoos in India^22^. Studies indicate that India’s surface temperature has risen by around 0.7 °C during 1901–2018^43^ and 1 °C during winter and post-monsoon months over the last century^44, 45^. There is a need to understand the burden on healthcare services due to heatwaves, which can be crucial in optimal planning, preventive activities, and policy-making, especially in low- and middle-income countries^21^. A Coupled Model Inter Comparison Project phase 5 (CMIP5) data analysis shows that heatwaves will be longer, more intense, and more frequent than earlier^46, 47^.

The critical infrastructure of the area also influences the impacts of extreme weather events. This includes public services like roadways, electricity, accessibility, and affordability of health and water supply^21^. Adaptive behaviors for combating heatwaves can be categorized in different ways, including education and awareness raising, adaptation of critical infrastructure, government measures, and health-related measures^48^. Socio-economic conditions, language barriers, neighborhoods, and occupations also influence the vulnerability to heat^49^. Climate change and its implications in extreme temperatures are a global public health threat. It is highly inequitable as poor developing nations are more vulnerable and have the least contribution to greenhouse gas emissions (GHGs)^50, 51^. Pollution and humidity would enhance the temperature effect^52^.

Heatwave occurrences are widespread across India, except for the west coast and northeast regions. There are two prominent areas where most heatwave events occur, i.e., Northwest and Southeast India^45, 53^. With the increase in global warming levels, most of India’s regions will face severe heatwaves. Still, the Himalayan, Coastal, and North-east regions are the most vulnerable to heatwaves and associated severity^54, 55^. Indian smart cities, particularly in the Coastal, Interior Peninsular, and North-Central regions, will observe intense and frequent heatwaves in the future under both moderate (SSP245) and high (SSP585) emissions scenarios^9, 10^. Under the SSP585 scenario, maximum temperatures are projected to rise by 4 °C and minimum temperatures by 5.5 °C by the end of the twenty-first century across northeast India and Bangladesh. The crisis is projected to worsen dramatically, with climate models predicting 128,000–259,000 additional annual deaths by mid-century under moderate to high warming scenarios^56^. The number of summer days is expected to increase by 53 days annually^57^, whereas in Chennai, over 100 heatwave days are expected annually by 2071–2100^58^. The Western Himalaya region (2.0–4.3 °C) shows the highest increase in intensity of 2-day duration, followed by the Northwest (0.93–2.51 °C) and Eastern Coastal (0.86–2.5 °C) regions under different global warming levels^54, 55^.

There is heterogeneity in mortality risks among urban areas, and the risk increases with intensity. According to occupational health studies, agricultural and outdoor workers bear the heaviest burden of heat mortality, facing 35 times higher death rates than other industries^59^. People in hotter environments are more susceptible to cold, and those in colder climates are more vulnerable to heatwave impact^60^. There is a declining severity of extreme cold events and an increase in warm events^61^. The human body has multiple thermoregulatory mechanisms to counter external extreme temperatures, the main objective of which is to keep temperature homeostasis within normal values. As exposure time to these stressful conditions increases and the external temperature becomes even more extreme, the body systems progressively adapt to its environment^62^. Studies show that the increase in ambient temperature leads to a rise in excess all-cause mortality^63, 64^. With over 24,000 deaths attributed to heatwaves from 1992 to 2015, there is an urgent need to understand India’s vulnerabilities and prepare adaptive strategies under various emission scenarios^43^.

However, most of these studies, which focus on assessing the effect of temperature extremes, were conducted in other parts of the world. There are very few Indian studies investigating the impact of extreme temperatures on overall mortality rates, predominantly in megacities like Ahmedabad^63, 65^ and Delhi^66^. Also, previous studies have been conducted based on theoretical modeling and were not based on the actual mortality data. Hence, this study aims to establish the linkage between the observed temperature and the associated mortality in Tier 2 city (here Chandigarh), which has not been explored. Therefore, we have attempted to study the influence of temperature extremes on all-cause mortality,to identify the duration and intensity of heatwaves beyond which mortality risk increases; to determine age- and gender-specific vulnerabilities; and to evaluate the excess mortality associated with these events.

## Methodology

To evaluate the impact of extreme temperature on daily all-cause mortality and to conduct a need-based assessment, we have adopted the following methodology:

### Definition of extreme temperature events

The definition of heat and cold waves varies across climate zones, based on temperature relative to local climatic conditions. For instance, the U.S. National Oceanic and Atmospheric Administration (NOAA) defines a heatwave as “a period of abnormally and uncomfortably hot and unusually humid weather”^67^, (Russo et al., ^68^). In contrast, the Indian Meteorological Department (IMD) uses a definition based on fixed temperature thresholds, which is more suitable for general climate monitoring. The IMD considers a heatwave when a station’s maximum temperature reaches at least 40 °C or more in the Plains and at least 30 °C or more in Hilly regions for two consecutive days. Their definition further categorizes heatwaves in two ways.

- Based on departure from normal: a Heatwave is declared when the temperature is 4.5–6.4 °C above normal, and a Severe Heatwave when it is > 6.4 °C above normal.
- Based on actual maximum temperature: a Heatwave occurs when the actual maximum temperature is ≥ 45 °C, and a Severe Heatwave when it is ≥ 47 °C.

To assess definitional sensitivity, we compared five heatwave criteria: the IMD threshold (≥ 40 °C for ≥ 2 consecutive days), the NOAA definition (consecutive days above the 90^th^ percentile of daily maximum temperature), and three percentile-based thresholds (90^th^ percentile: 41.2 °C, 95^th^ percentile: 42.8 °C, and 99^th^ percentile: 44.1 °C). The 90^th^ percentile was calculated from the local temperature distribution for 2010–2015.

A cold wave is a weather condition that is distinguished by the cooling of the air. The IMD definition considers the wind chill factor; the wind chill factor plays a vital role in reducing the actual minimum temperature depending on the wind speed^45^. At any location with a normal temperature above 10 °C, a decrease of − 5 to − 6 °C below normal is considered a cold wave. For any location with a normal temperature below 10 °C, a reduction of − 4 to − 5 °C is regarded as a cold wave (India Meteorological Department, ^69^).

### Study area

The study was carried out in Chandigarh. It is a city and union territory serving as Haryana and Punjab’s capital. It’s a well-planned city. It was planned by the French architect Le Corbusier. A Regional Meteorological Centre is also located in Chandigarh. Figure S1 shows Chandigarh’s location (30.7°N, 76.8°E). The city borders Punjab and Haryana, serving as a joint capital. Its Köppen-Geiger classification (Cwa) represents it has a hot summer, cold, dry winter, and sub-tropical monsoon. The weather is dry, and evaporation is greater than precipitation.Chandigarh was strategically selected because it is located in the climatically vulnerable Indo-Gangetic Plain experiencing rapid warming, and serves as a best example of India’s tier 2 urban planned city with organized sectors and above 30% green cover, union territory status which ensures superior data quality in death registration completeness and experiences extreme temperature range (4–46 °C) capturing full temperature-mortality spectrum.

### Data collection

The daily all-cause mortality data and daily meteorological data were collected from the Office of the Registrar of Birth and Death and the Regional Meteorological Center, Chandigarh, respectively, for 6 years, from 2010 to 2015. Permission was obtained from the Director of Health and Family Welfare, Chandigarh Administration, via letter number MH-II-20171/14,278 dated 27^th^ June 2017, to collect daily all-cause mortality. The data includes the daily all-cause mortality, age, gender, and place of death of the deceased. For our study, we included those who were residents of Chandigarh. All experimental protocols were approved by the Institutional Ethics Committee, Postgraduate Institute of Medical Education and Research, Chandigarh vide letter number INT/IEC/2017/476, dated 2^nd^ May 2017, and all experiments were performed in accordance with relevant guidelines and regulations. The ethics committee waived the requirement for informed consent.

The meteorological data includes half-hourly temperature values for 24 h a day. There are a total of 48 values for a day. A key assumption of our study is that the analysis is conducted using data from a single station due to Chandigarh’s relatively small geographical area and spatial homogeneity. This study used an ecological study designed to assess the excess all-cause mortality.

### Data analysis

The relationship between temperature and mortality was established using the over-dispersed Poisson Generalized Additive Model (GAM)^36^. According to this model, mortality rate and temperature are related as follows:

1 $$\log E\left( {Y_{t} } \right) = \beta_{0} Z_{t} + DOW + ps\left( {time_{t} ,4} \right) + ps\left( {Temp,4} \right)$$

Where

${Y}_{t}$= expected mortality rate for city c on day t;

$${\beta }_{0}$$= model intercept;

${Z}_{t}$ = indicate the visibility.

${DOW}_{t}$= categorical variable for a day of the week;

ps (${time}_{t}$) = penalized spline of time, with 4 degrees of freedom (df);

ps(Temp)= penalized spline of a temperature metric for city c for day t, with 4 df

The temperature-mortality response curve was estimated, and the heat and cold-related portion of the relationship was summarized by evaluating the effect of a change in absolute and relative temperature. To determine the impact of relative temperature change, a change in mortality risk was calculated by comparing the 1^st^ to 10^th^ percentile for the cold effect and the 99^th^ to 90^th^ percentile for the heat effect of the temperature distribution^36^.

The visibility factor was considered and used as a surrogate for other meteorological variables to assess the effect of extreme heatwave conditions on mortality. The above GAM model was used to study the temperature mortality response. The heatwave was categorized using the IMD criteria. Mortality rate ratio analysis was done by applying the methodology of Lan et al.^70^ by taking the extreme heat period (H) and a reference period (R)^63, 71^. Correlation coefficients for mortality and temperature were calculated between daily maximum temperature and daily all-cause death count using monthly correlations^63^.

The above-explained GAM model is used to determine the effect. All analyses were conducted in R using *mgcv* and *gamair* packages (R 3.4.1 development core team) and SPSS. In the GAM, we took the link as a log function, and the Poisson Family was chosen because we saw that mortality follows the Poisson distribution. We compared the over-dispersed Poisson GAM with a Distributed Lag Non-Linear Model (DLNM) implemented using the dlnm package^20^. The DLNM specification included a natural cubic spline with 3 df for temperature and 4 df for lag (0–7 days), allowing for non-linear and delayed associations. Model comparison used Akaike Information Criterion (AIC), Deviance, and mean squared error (MSE).

A segmented regression (also known as breakpoint or piecewise regression) analysis was performed to statistically identify the optimal temperature threshold for increased mortality. This approach systematically tests a range of temperature thresholds (50 equally spaced values between the 10^th^ and 90^th^ percentiles of the temperature distribution) and, for each candidate threshold, fits a model that estimates mortality based on a linear relationship below the threshold and a different, steeper linear relationship for temperatures above it (the “excess” temperature). The optimal threshold is then selected as the temperature value that yields the most statistically significant (lowest *p*-value) and positive coefficient for the excess temperature variable, indicating a point beyond which each additional degree is associated with a substantial increase in mortality.

Lagged associations were assessed using distributed lag models with a maximum lag of 7 days. Cross-basis functions combined natural cubic splines (3 df) for temperature and lag dimensions.

## Results

### Demographic characteristics of the daily all-cause mortality data

The daily all-cause mortality data for each month from 2010 to 2015 was analyzed. While the total number of days for analysis was 2191, data for 36 days was unavailable, resulting in a total count of 2155 days. The data was manually collected and compiled from the Department of Birth and Death Registrar, comprising records of every death that occurred in Chandigarh during that period.

### Gender and age-standardized daily all-cause mortality

**Fig. 1:**
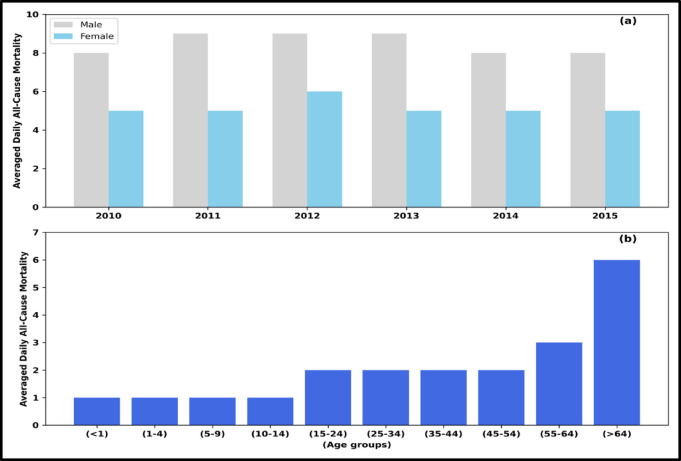
Averaged daily all-cause mortality rates by (**a**) gender and (**b**) age-standardized computed in Chandigarh, India.

For age-standardized, average all-cause mortality per day, we divide each group’s total daily all-cause mortality by the total number of days. The age standardization of daily all-cause mortality shows that the maximum number of deaths has occurred in the age group of (> 64), with nearly an average of 6 deaths per day. If we take the groups for ages less than 25, the daily all-cause mortality is higher for ages less than or equal to 1 but decreases with the groups (< 4), (10–14), and (15–24), respectively. Further mortality increases down the group for age groups above 25, that is, (25–34), (35–44), (45–54), (55–64), and (> 64), respectively. Averaged daily all-cause mortality rates by gender and age-standardized are shown in Fig. 1. The results show that males’ average daily all-cause mortality is more than that of females. The average daily all-cause mortality for males is 9, and for females, it is 6. The maximum number of deaths occurred in the year 2012, which was 5386, with an average of 15 deaths per day. The minimum number of deaths occurred in 2015, which was 4579, with an average of nearly 12 deaths per day.

### Descriptive statistics for the time (day) mean temperature, visibility, and daily all-cause mortality

From the analysis of 2155 days of daily all-cause mortality data, the average number of deaths was found to be 14 per day. Additionally, the median daily all-cause mortality ranged between 11 and 17 deaths, with an interquartile range of 6. The maximum mortality for any day is 38, and the minimum mortality was observed as 0. The total number of values evaluated for mean daily temperature is 2191. The minimum value of the mean daily temperature is 4 °C, and the maximum value of the mean daily temperature is 38 °C. The 50 percent of the values for mean daily temperature lie between 17 and 29 °C, the interquartile range for which is 21 °C.

### Temperature mortality response estimation

The relationship between temperature and mortality was established using the over-dispersed poisson generalized additive model (GAM), as shown in Eq. 1. According to this model, mortality rate and temperature were found to be associated, as discussed below:

The visibility factor was also considered and used as a surrogate for other meteorological variables to assess the effect of extreme heatwave conditions on mortality. It was observed from the output of the above model that visibility and temperature significantly affect the daily mortality of the city. The R^2^ describes the extent to which the prediction model fits the data. From the value of R^2^, which is 0.996, we can see that it is very close to one, and the model explains 99.1% of the variation. Figure S2 depicts the residuals vs. the linear predictor, showing that the model performs well. Also, the curve for Response *vs*. Fitted value, we can interpret that a linear trend is coming out. We can conclude from the graph that the model fits the data well and can be used to predict future values. It can also be seen from the model that the unbiased risk estimated score is 2.87.

An over-dispersed Poisson GAM based on over-dispersion of mortality data was used. We have also used an alternative modeling approach bydirectly comparing the standard over-dispersed Poisson GAM and a Distributed Lag Non-Linear Model (DLNM) as shown in Fig. S3 framework. Our analysis revealed that while the DLNM approach, which accounts for delayed temperature effects on mortality, offers a more biologically plausible structure for capturing distributed lag patterns, the standard GAM demonstrated superior performance in our specific dataset. The GAM achieved better fit statistics with a lower AIC (2062.23 vs 2067.17) and lower mean squared error (15.35 vs 16.08), suggesting that for this particular temperature-mortality relationship, the GAM’s ability to capture non-linear exposure–response relationships without explicit lag structures provided more precise risk estimates. However, we acknowledge that the DLNM framework remains valuable for understanding the temporal dynamics of heat effects, and we will incorporate it as a sensitivity analysis to ensure our risk estimates are robust across different modeling approaches, particularly for examining the distributed nature of heat-related mortality impacts.

The mean mortality is ~ 12.7 in summer and in winter it is 15.80, while the variance is ~ 17.5 in summer and 23.65 in winter. The dispersion statistic is ~ 1.34 in summer and ~ 1.47 in winter, as shown in Fig. S6. Since variance is higher than the mean, this suggests over-dispersion in the data in both seasons. Over-dispersion testing confirmed variance exceeded mean for both seasons (summer: φ = 1.34; winter: φ = 1.47), violating standard Poisson assumptions. The quasi-Poisson GAM with scale parameter estimation accommodates this over-dispersion while maintaining interpretability. Alternative approaches considered included: negative binomial regression (rejected due to convergence issues), zero-inflated models (unnecessary given only 2% zero-mortality days), and quasi-likelihood methods (inferior AIC).

Also, it is noticed from Fig. S2 of response *vs.* fitted values that there was an apparent fit of the values, and a straight-line fit of the model was formed. The histogram of residuals shows a positive skew, indicating that with the increase in temperature, there was the possibility of an increase in mortality. The heavier tails in the residual distribution indicate that the model underestimates the probability of extreme events, representing a key limitation for predicting outlier values.

### Relationship between temperature and mortality

**Fig. 2:**
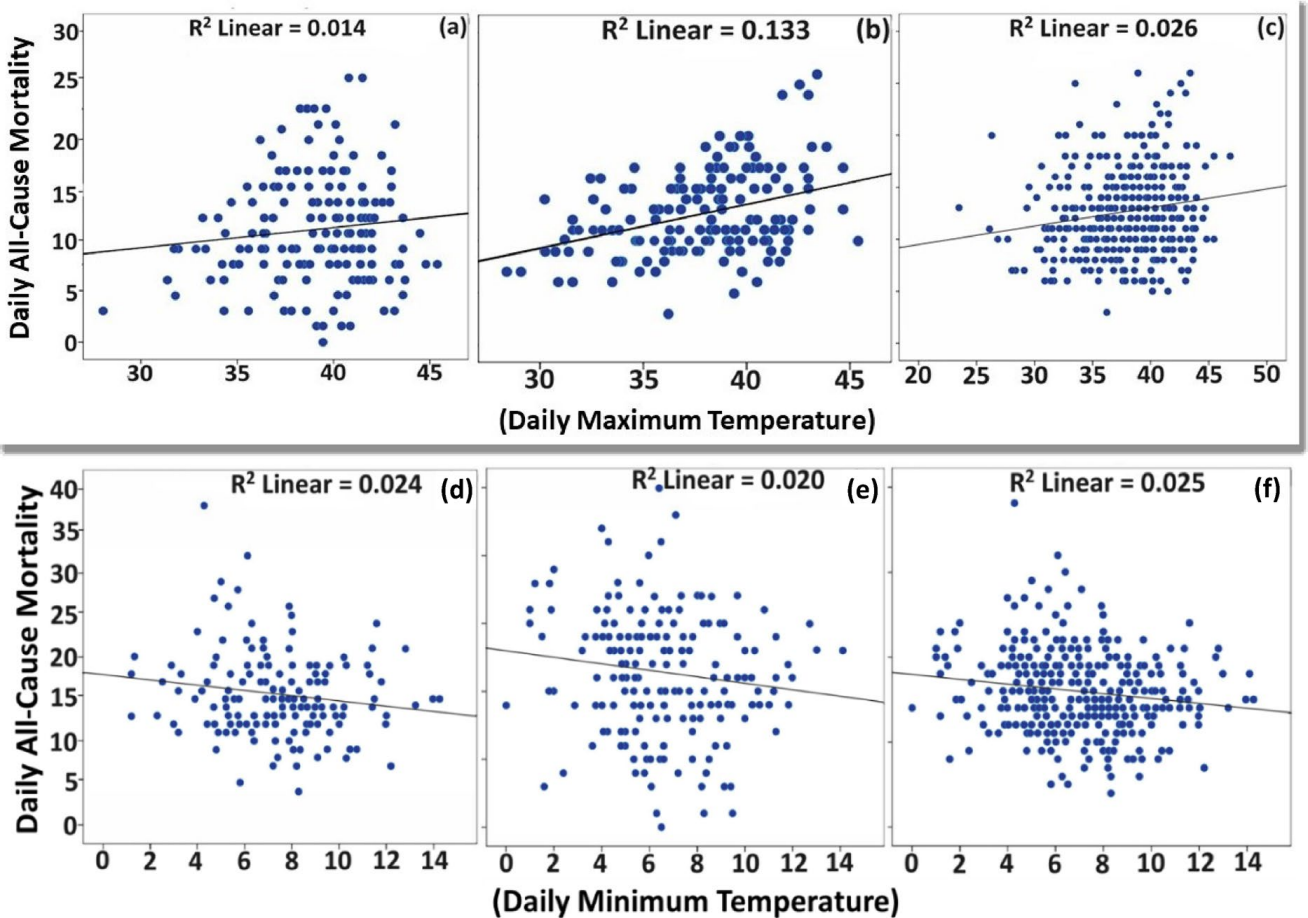
Scatter plot of (**a**) May, (**b**) June, and (**c**) averaged daily all-cause mortality vs daily maximum temperature (above), and scatter plot of (**d**) December, (**e**) January, and (**f**) averaged daily all-cause mortality vs daily minimum temperature (below).

**Fig. 3:**
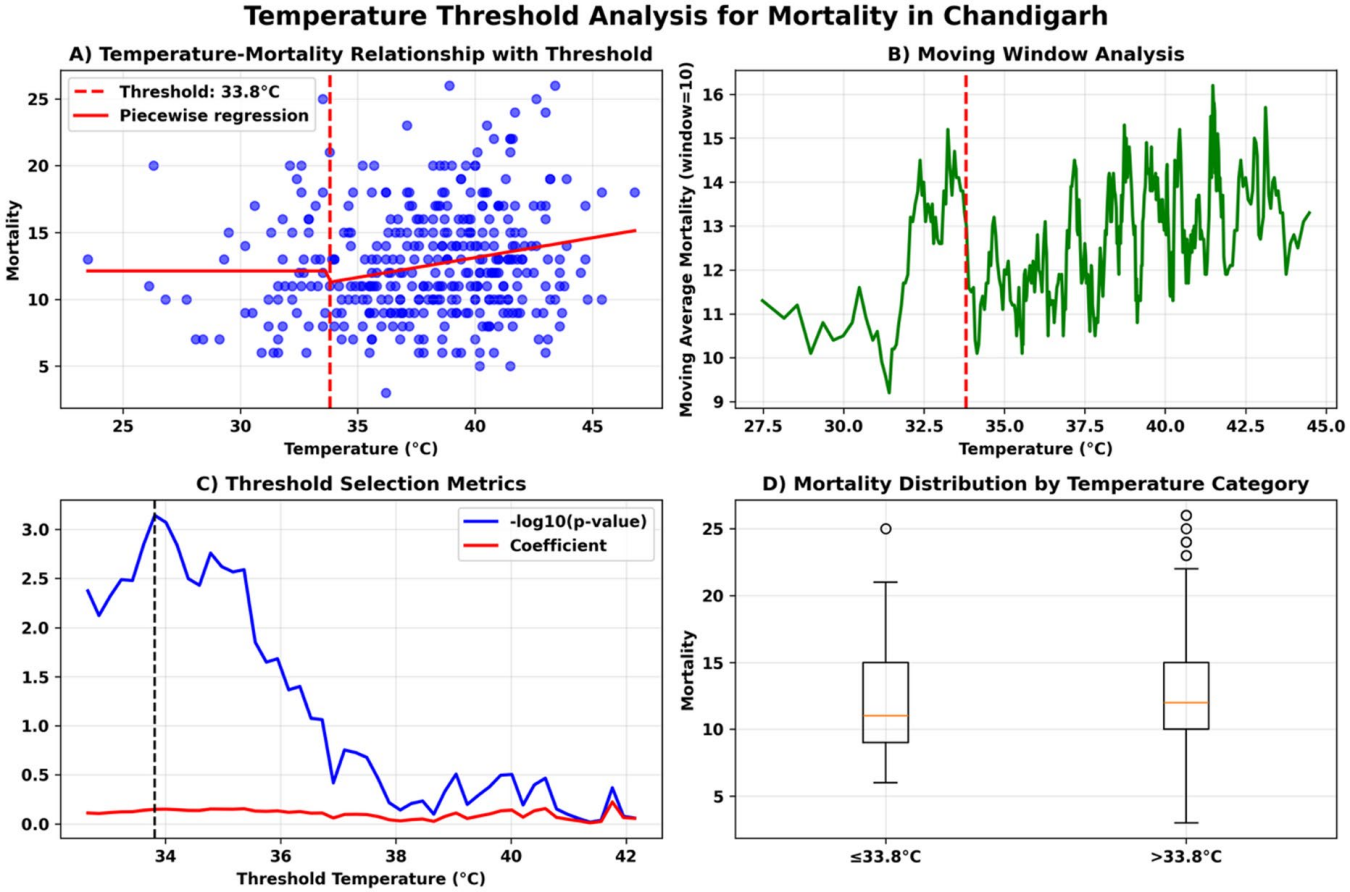
Temperature threshold analysis for mortality at Chandigarh (**a**) Temperature-Mortality Relationship with Threshold (**b**) Moving window analysis (**c**) Threshold Selection Metrics (**d**) Mortality Distribution by Temperature Category.

Monthly correlations were established for daily maximum temperature and daily all-cause mortality counts for hot weather months, May and June, and cold weather months, December and January, as shown in Fig. 2. The correlation coefficient values show significant variation over the considered period. The r value for May in 2010 shows almost no correlation, whereas it is practically perfect (r = 0.98) in 2012. The maximum value for r was 0.98 for May 2012, suggesting a substantial positive value. The minimum r value was 0.039 for May 2015, indicating no correlation. For June, the minimum value was 0.01 in 2014, and the maximum was 0.55 in 2012. For the winter months, the correlation value was negative, suggesting an increase in mortality with a decrease in temperature. The minimum value for January was (− 0.065), and the maximum value was (−0.80). For December, the minimum value was (− 0.023), and the maximum value was (0.26), indicating a weak correlation (Fig. 3).

**Fig. 4:**
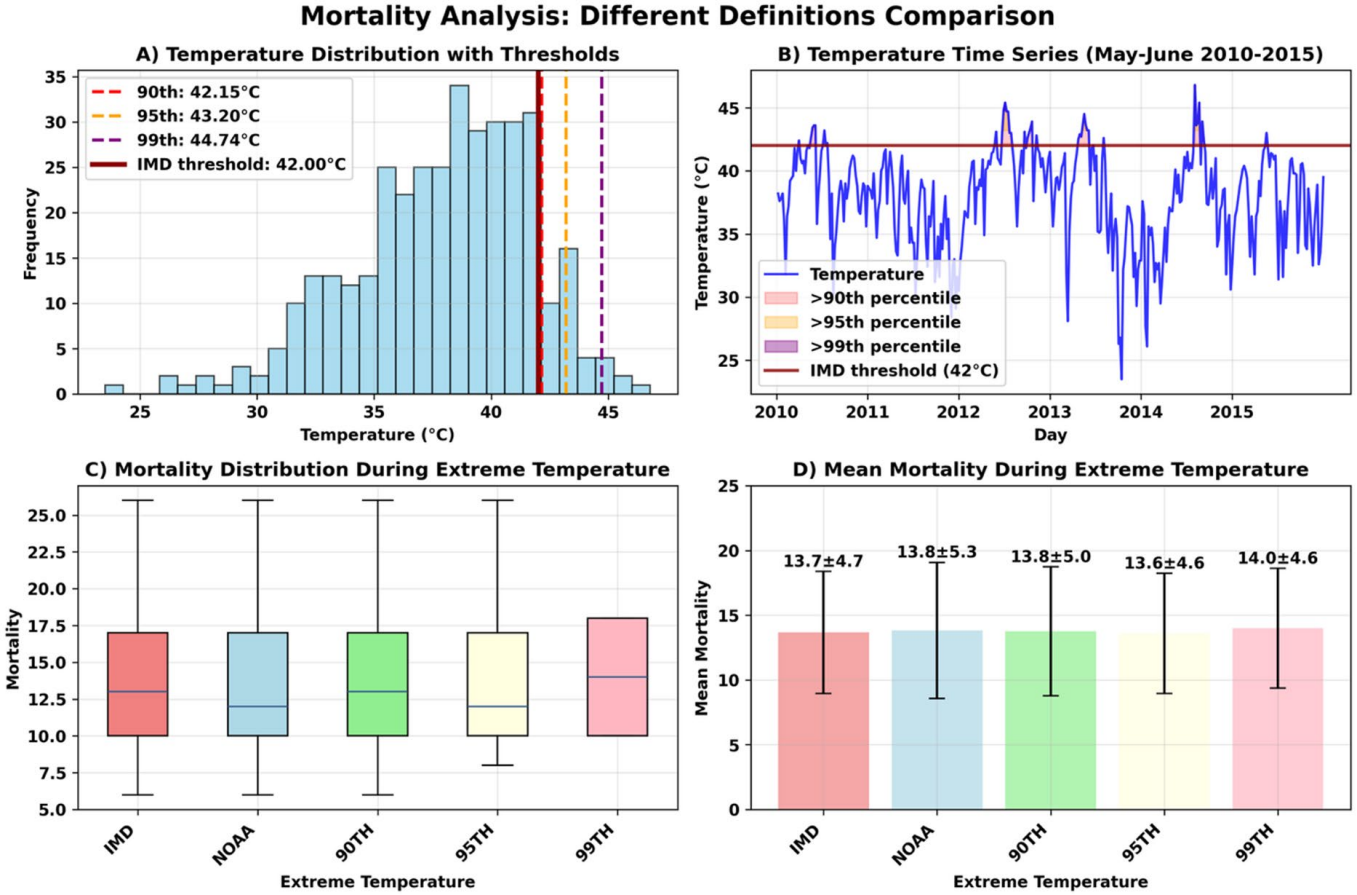
Mortality estimation as per definitions of heatwaves (IMD vs NOAA vs percentile thresholds) (**a**) temperature distribution with thresholds (**b**) Temperature time series (**c**) Mortality distribution during extreme temperature (**d**) Mean Mortality during extreme temperature.

The choice of heatwave definition significantly influences mortality estimation outcomes in this analysis. We compared five different methodologies: the IMD threshold (≥ 42 °C for at least one day), the NOAA definition (consecutive days above the 90^th^ percentile), and percentile-based thresholds (90^th^, 95^th^, and 99^th^ percentiles) as shown in Fig. 4. The IMD definition identified a substantial number of extreme temperature days, with 42 days, while progressively stricter definitions captured fewer events: 37 days at the 90^th^ percentile, 29 days using NOAA criteria, 17 days at the 95^th^ percentile, and only 4 days at the 99^th^ percentile.

All definitions showed higher mortality during extreme temperatures, with mortality ratios ranging from 1.07 to 1.10, reflecting a 7–10% increase compared to non-heatwave days. However, in this analysis, none of the definitions produced statistically significant results (p-values ranging from 0.1732 to 0.6192). This lack of significance is likely due to limited statistical power from smaller sample sizes rather than a lack of true effect. While the IMD definition captures the largest number of events, its results were not statistically significant in this dataset. The consistency of elevated mortality ratios across all definitions suggests a measurable impact, but larger sample sizes are needed to confirm statistical significance.

To examine whether the duration of a heatwave affects the risk of death, we analyzed mortality during IMD-defined heatwaves of varying lengths (≥ 1, ≥ 2, ≥ 3, and ≥ 5 consecutive days with temperatures ≥ 42 °C) as shown in Table 1. The results indicate that mortality during heatwaves is generally higher than on non-heatwave days, but the differences are not statistically significant. Specifically, IMD ≥ 1-day heatwaves had 42 days with a mortality ratio of 1.08 (*p* = 0.1732), IMD ≥ 2-day heatwaves had 35 days with a similar mortality ratio of 1.08 (*p* = 0.2542), IMD ≥ 3-day heatwaves had 31 days with a slightly higher ratio of 1.10 (*p* = 0.1936), and IMD ≥ 5-day heatwaves had 15 days with a near-null ratio of 1.01 (*p* = 0.9590). These findings suggest that, under the IMD definition, heatwave duration does not significantly influence mortality risk.

**Table 1:** Heatwave duration and associated risk of death.

Heatwave duration	Heatwave days	Mortality during heatwaves (Mean ± SD)	Mortality non-heatwaves (Mean ± SD)	Mortality ratio	Mortality difference
≥ 1 day (IMD_1day)	42	13.67 ± 4.71	12.61 ± 4.10	1.08	1.05
≥ 2 days (IMD_2days)	35	13.66 ± 5.04	12.64 ± 4.08	1.08	1.02
≥ 3 days (IMD_3days)	31	13.90 ± 5.21	12.63 ± 4.07	1.10	1.28
≥ 5 days (IMD_5days)	15	12.80 ± 4.95	12.73 ± 4.16	1.01	0.07

To further evaluate the relationship between temperature extremes and mortality, as shown in Fig. S5, we categorized days into different IMD-defined temperature classes relative to the normal threshold of 38 °C. During normal temperature days (n = 209), mortality averaged 12.27 ± 3.99, with a statistically significant *p*-value (0.0159). In contrast, mortality increased modestly during hotter categories but did not reach statistical significance. Specifically, above normal days (n = 36) and appreciably above normal days (n = 83) both recorded a mean mortality of 13.22, while moderate heatwave days (n = 31) showed 13.71, and severe heatwave days (n = 7) reached 14.00. These findings suggest that mortality rises with increasing temperature categories, but only on normal days exhibited a significant difference.

#### Relationship between mortality and temperature for hot weather months

The coefficient of determination (R^2^) with *p*-value is derived to determine the strength of the linear relationship between the daily maximum temperature and daily all-cause mortality. A scatter plot of daily all-cause mortality and daily maximum temperature in May is shown in Fig. 2a. The value of May’s coefficient of determination (R2) was 0.014. As the value was less than 1 but greater than 0 and was positive, mortality increased as the temperature increased, but the correlation is weak and statistically significant (*p* < 0.05). It was observed from the scatter plot that there was an increase in mortality with the increase in temperature. The maximum numbers of deaths were clustered around the temperature of 40 °C. There are fewer values beyond the temperature of 42 °C and before 37 °C or 38 °C.

The linear relationship for June shows a moderate positive relationship between the daily maximum temperature and the daily all-cause mortality. A scatter plot of daily all-cause mortality and daily maximum temperature in June is shown in Fig. 2b. The R^2^ value was 0.133 and is statistically significant with a *p*-value of 0.000. There was an increase in mortality with an increase in temperature. The coefficient of determination is positive but more than 0, which means there is a linear relation between the all-cause mortality and the daily maximum temperature of May and June (2010–2015). Daily mortality will increase with the temperature increase, but the increase is slight. The linear relationship between averaged daily all-cause mortality and maximum temperature was weak, as shown in Fig. 2c. That is, the impact of the increase in temperature is not that strong. As the *p*-value is less than 0.05, it’s statistically significant.

Our analysis identified a statistically significant temperature threshold of 33.8 °C (*p* = 0.0007) above which mortality increases in Chandigarh, though the absolute effect size is modest. The practical impact is relatively small as mortality increases by only 4.1% (from 12.30 to 12.81 deaths per day) with an attributable risk of 0.509 additional deaths, as shown in Fig. 3.

#### Relationship between mortality and temperature for cold weather months

An inverse relationship exists between temperature and mortality variable for winter. The correlation coefficient (r) value for December is negative, and mortality will increase with the decrease in temperature. A scatter plot of daily all-cause mortality and daily minimum temperature in December is shown in Fig. 3a. The value for r =  − 0.155 (R^2^ = 0.024), which is less than 0, means a weak inverse linear relationship exists between daily all-cause mortality and daily minimum temperature mortality. The *p*-value is 0.027, less than 0.05. Although there is a weak inverse relationship, it is statistically significant.

Daily all-cause mortality and daily minimum temperature of January (2010–2015) analysis shows an inverse relationship between the temperature and mortality variable for winter months. A scatter plot of daily all-cause mortality and daily minimum temperature in January is shown in Fig. 3b. The r value for January is negative, which means mortality will increase with the decrease in temperature. The value for r =  − 0.141 (R^2^ = 0.020), which is less than 0, indicates that a weak inverse linear relationship exists between daily all-cause mortality and daily minimum temperature mortality. The *p*-value is 0.028, less than 05. Although there is a weak inverse relationship, it is statistically significant.

There is an inverse relationship between the temperature and mortality variables for January and December (2010–2015). Evidence from a range of disciplines appears to support a causal relationship for cold across a range of temperatures and lag periods, although there is more consistent evidence for a causal effect at more extreme temperatures^72^ The r value for the total daily all-cause mortality of January and December concerning daily minimum temperature is negative, which means mortality will increase with the decrease in temperature. The value for r =  − 0.158 (R^2^ = 0.025), which is less than 0, indicates a weak inverse linear relationship exists between daily all-cause mortality and daily minimum temperature mortality. A scatter plot of daily all-cause mortality and daily maximum temperature in May is shown in Fig. 3c. The *p*-value is 0.002, less than 0.05, although it is a weak inverse relationship and statistically significant.

### Heatwave and associated excess all-cause mortality

We categorized the data into heatwave days by using the IMD criteria. For any station where the normal maximum temperature is less than or equal to 40 °C, the heatwave departs from normal temperature by 5–6 °C, as shown in Table 2. For Chandigarh, the normal maximum temperature is 38 °C. We counted days with temperatures equal to 42 °C and above as heatwaves. We compare the daily all-cause mortality on these days with the daily all-cause mortality of similar periods of succeeding and preceding years.

**Table 2:** Heatwave and associated excess all-cause mortality in Chandigarh, India.

S.no	Day	Duration	Max. (°C)	Min. (°C)	Daily mortality	Reference year mortality	Excess mortality	Percent excess
1	24–26 May 2012	3	42	27	16	14	2	14.2%
2	29–31 May 2012	3	44.3	25.1	19	11	8	63.6%
3	1–3 June 2012	3	44.1	29.5	19	15	4	26%
4	15–17 June 2012	3	43	30.8	17	12	5	41.6%
5	20–21 June 2012	2	42.25	29.2	21	10	11	100%
6	29–1 July 2012	3	41.5	29.33	21	14	7	50%
7	19–26 May 2013	7	43.2	26	15	13	2	15%
8	31 May 2014	1	43.5	23.5	13	11	2	18%
9	5–11 June 2014	8	44.2	26.8	15	12	3	25%

It was observed from Table 2 that there was an increase in mortality during the days of heatwaves. However, the variation was present in an increase in mortality, and the excess was different for different periods, depending on various other factors that also affect the mortality. Maximum excess mortality was seen during the 20^th^ and 21^st^ June of 2012, which is 100% excess mortality. Minimum excess mortality was seen on 23^rd^ to 26^th^ May, which was 14.2%. The most extended duration of heatwave days was from 5 to 11^th^ June 2014, when there was a 25% increase in mortality. The shortest duration of a heatwave day was 31^st^ May 2014, when there was 18% excess daily all-cause mortality.

### Mortality rate ratio analysis for the heatwave period

The mortality rate ratio analysis for the heat wave period for the years 2012 and 2013 using the methodology adopted by Lan et al.^71^ and Azhar et al.^63^. We categorized the heatwave period according to IMD criteria. We compared the daily all-cause mortality during the heatwave period (H) with reference values, as shown in Table 3. We took 2013 as the reference year for 2012 and 2014 as the reference year for 2013. As the heatwave period from 24^th^ May to 26^th^ May 2012 is almost similar to the 19^th^ May to 27^th^ May 2013, we took the reference year 2011 as the period considered in 2012. We calculated the confidence interval by using the formula.

**Table 3:** Mortality rate ratio analysis for the heatwave period during (2012–2013).

S.no	Day	Daily mortality (H)	Reference year mortality (R)	Risk ratio (RR)	95% confidence interval
1	24–26 May 2012	16	14	1.142	0.55–2.74
2	29–31 May 2012	19	11	1.72	0.821–3.62
3	1–3 June 2012	19	15	1.26	0.64–2.49
4	15–17 June 2012	17	12	1.41	0.67–2.96
5	20-21June 2012	21	10	2.1	0.98–4.45
6	29–1 July 2012	21	14	1.5	0.76–2.94
7	19–26 May 2013	15	13	1.153	0.54–2.42
8	31 May 2014	13	11	1.18	0.52–2.63
9	5–11 June 2014	15	12	1.125	0.58–2.67

2 $$CI=\mathrm{exp}(logRR\pm 1.96\sqrt[2]{\frac{1}{H}+1/R)}$$

The formula used for the mortality rate ratio was

3 $$RR=H/R$$

It has been observed from the analysis that mortality risk increased considerably during the heatwave period. The mortality risk almost doubled during the two-day heatwave period of 20^th^ -21^st^ June 2012. The average value for the risk ratio is 1.41, with a mean confidence interval of (0.675–2.95). We compared the daily mortality value for the heatwave and reference periods according to Eq. 2. The average risk ratio was observed to be almost the same for males and females, 1.40 and 1.48, respectively. The mortality risk ratio value shows a variation for different periods. There might be other factors involved that also influence the mortality. On 24^th^–26^th^ May 2012 and 29^th^ June–1^st^ July, RR is 1 for females, which means there is no change in mortality. There is considerable variation in value, with a standard deviation of 0.51 and 0.81 for males and females, respectively. The gender-specific mortality rate ratio calculation for the heatwave period is shown in Table 4.

**Table 4:** Gender-specific mortality rate ratio calculation for the heatwave period.

S.no	Day	Daily mortality (H)		Reference year mortality (R)		Risk ratio (RR) (M)	Risk ratio (RR) (M)
		M	F	M	F		
1	24–26 May 2012	23	14	24	13	0.95	1
2	29–31 May 2012	25	8	19	17	1.31	0.47
3	1–3 June 2012	36	31	35	9	1.02	3.44
4	15–17 June 2012	42	20	29	14	1.44	1.85
5	20–21 June 2012	28	13	24	7	2.44	1.38
6	29–1 July 2012	44	18	18	13	0.86	1
7	19–26 May 2013	59	39	68	39	2	0.778

## Discussion

Our study found a significant excess of all-cause mortality associated with the heatwave. Temperature mortality response analysis is done using a Poisson General Additive Model (GAM), to account for non-linear relationships between temperature and mortality (Wood, ^73^; [Ravindra et al., 2019]). We have used visibility as a surrogate for other meteorological factors, as per the approach of Huang et al.^74^ in their Shanghai study, in which the model’s output showed that visibility and temperature significantly affected the daily mortality of the city. The model has shown high goodness of fit, with a value of R^2^ of 0.996. We can see that it is very close to one, and the model explains 99.1% of the variation occurring in mortality.

The heatwaves were categorized based on the IMD criteria. To consider a heatwave, the maximum temperature of the station should be at least 40 °C for plains and 30 °C for hills. For Chandigarh, the normal maximum temperature is 38 °C, so according to IMD criteria, when the normal maximum temperature is less than or equal to 40 °C, the heatwave is considered as the departure from normal by 5–6 °C. In our study, the days with temperatures of 42 °C and above are considered heatwave days. A severe heatwave is considered when there is a departure from normal by 6–7 °C.

There were, in total, 33 heatwave days that also included one severe heatwave day of 45.4 °C on 31^st^ May 2012. These heatwave days were taken for 2012 and 2014 to compare them with the average mortality of the succeeding and preceding years. Our study shows an increase in all-cause mortality associated with heatwave days, which is consistent with the findings of other studies. A survey conducted in Gujarat on mortality during the 2010 heatwave found a 43.12% increase in daily all-cause mortality^63^. Twenty-three analyzed multiple heat events for 2012, 2013, and 2014, comparing the daily all-cause mortality with the averaged reference year mortality. We find variation to be as high as 100%, as seen on 20^th^–21^st^ June 2012, and the lowest to be 14.2% on 24^th^–25^th^ May 2012.

Age-stratified analysis revealed that individuals over 65 years had a 1.5 times higher risk of heat-related mortality compared to the general population (Fig. 1). We observed substantial variability in gender-specific mortality risk, with risk ratios ranging from 0.86 to 2.44 for males and 0.47 to 3.44 for females. However, mean risk ratios were similar for both genders (males: 1.43, SD = 0.59; females: 1.42, SD = 0.98). Although the variation in the excess all-cause mortality can be explained by studying other factors that can modify the excess all-cause mortality, potential confounding factors include air pollution, PM_10_, PM_2.5_, and humidity. Some previous studies have shown that temperature considerably affects mortality, independent of air pollution^36, 63^. A study conducted in Western Europe showed that an association was present between particulate air pollution and temperatures. However, there is a stronger association between air particulate matter and mortality in summer than in winter^74^. We did not include these factors in our analysis.

We also studied the monthly correlation between maximum temperature and daily all-cause mortality for summer months. Our finding suggests a moderate to weak relationship between mortality and temperature. The observed negative correlation between minimum temperature and mortality in winter months (r =  − 0.158, *p* < 0.01) suggests a cold-related mortality effect consistent with findings from other cold regions^75^. If we see the year-wise correlations, the maximum for May 2012 was r = 0.98 (*p* < 0.001). This is also consistent with our finding that the maximum number of heatwaves were reported during that period, and one severe heatwave with a maximum temperature of 45.4 °C. It was observed from the correlation analysis that there was an increase in mortality with the increase in temperature. The correlation coefficient values were different for May and June; the values for June were more positive than those for May. The findings suggest a moderate correlation between daily maximum temperature and all-cause mortality. In a study conducted in Ahmedabad studying the effect of the 2010 heatwave, the correlation values show a strong positive relation between temperature and mortality of 0.775^63^.

Our findings for winter months showed a statistically significant negative correlation between excess daily all-cause mortality and daily minimum temperature (r =  − 0.158, *p* < 0.01). This inverse relation indicates an increase in mortality with a decrease in temperature. Our findings suggest that the continuous period of extreme heat presents an elevated risk over single days of high temperature, even if the heatwave period is as short as 2 days. During the most severe heatwave (20^th^–21^st^ June 2012), the relative mortality risk was 2.1, with a maximum temperature of 42.25 °C compared to non- heatwave days. In contrast, its mortality rate ratio is 1.18 for the single-day heatwave on 31^st^ May 2014 with a maximum temperature of 43.5 °C. This shows that duration and intensity of heat have an impact on the risk.

Lag analysis revealed primarily an immediate mortality response, as shown in Fig. S6. Same-day exposure showed the strongest association (RR = 1.09, *p* = 0.018), with rapidly diminishing effects at lag 1 (RR = 1.01, p = 0.561), lag 2 (RR = 0.99, *p* = 0.496), and lag 3 (RR = 1.00, *p* = 0.764). Cumulative 0–3 day exposure marginally exceeded same-day effect (RR = 1.11 vs 1.09), suggesting limited mortality displacement. The immediate response pattern supports our primary GAM specification, which focuses on the same-day temperature.

It is well understood that mortality is not only an outcome of exposure to temperature extremes but also a function of inherent vulnerability, such as age, pre-existing disease, poverty, low education levels, and air conditioning. The underlying assumption is that short-term temperature variation is unlikely to be correlated with other causes of death. Therefore, mortality variation on extremely hot or cold days will likely be linked to temperature. This study revealed excess all-cause mortality associated with extreme temperature events. The mortality rate varies with temperature changes. However, there is a need to evaluate other factors that might also influence daily all-cause mortality.

## Limitations

This study has considered only the effect of temperature on mortality; there is a need to study other environmental factors, like particulate matter and ozone levels, to come to a more effective conclusion. We stratified our daily all-cause mortality data by age and gender. To get a deeper insight into vulnerability, there is a need to consider other factors like socio-economic status and location. In developing countries like India, most of the deaths are unreported. Therefore, our results might be an underestimation of the real picture. Death records are in the process of digitalization, so manual analysis is time-consuming. The finding also requires careful consideration when comparing it with other Tier 2 cities because of Chandigarh’s unique characteristics. Chandigarh is a planned city with over 30% green cover sector organized in a rectangular pattern, due to which the city may experience lower heat vulnerability than other unplanned cities with similar climate characteristics of the Koppen Cwa/Cwg climate zone in the Indo-Gangetic plains and other populated areas of India.

## Conclusion

This study found a significant association between extreme temperature events, particularly heatwaves, and excess all-cause mortality in Chandigarh, India. The temperature-mortality response analysis using a generalized additive model (GAM) demonstrated that temperature and visibility significantly influenced daily mortality rates. During the study period, 33 heatwave days were evaluated, including one severe heatwave day with a maximum temperature of 45.4 °C. Our findings revealed a substantial increase in all-cause mortality associated with these heatwave days, consistent with previous studies. The excess mortality ranged from 14.2% to as high as 100%, highlighting the significant impact of heatwaves on mortality. The analysis of monthly correlations between maximum temperature and daily all-cause mortality for summer months suggested a moderate to weak relationship. However, the correlation was more decisive in specific months, such as May 2012, which matched the highest number of heatwave days and severe heatwave events. Overall, the present study indicated that the continuous duration and intensity of heatwaves play a crucial role in elevating mortality risks, with more extended and more intense heatwave periods posing a higher risk than single-day heatwave events.

While this study focused on the direct effects of extreme temperatures on mortality, it is crucial to recognize that mortality is influenced by various factors beyond temperature exposure, including age, pre-existing health conditions, socio-economic status, and access to air conditioning. To strengthen India’s heat action plans, real-time mortality data must be incorporated to enable us to detect heat health impacts. Despite some limitations, this study contributes to the growing body of evidence on the adverse health impacts of extreme temperature events in India. The findings underscore the urgent need for adaptation strategies which are enhanced using social economic indicators such as occupation housing type and income, public health interventions can be planned at even at finer level such as ward level to identify vulnerability hotspots, and policy measures to mitigate the adverse effects of climate change and protect vulnerable populations from the detrimental consequences of heatwaves and other extreme temperature events.

## Supplementary Information

Supplementary Information.

## Data Availability

The datasets generated and/or analyzed during the current study are not publicly available and were procured from the Office of Registrar Birth and Death, Chandigarh, India, only on request. But can be made available by the corresponding author at a reasonable request.
