# Supplementary material for: Extreme temperature events and their relationship with excess all-cause mortality in Chandigarh, India
Source: Sci Rep. 2026 Jan 29;16:4113. doi: 10.1038/s41598-025-32614-6 (PMC12859097; doi:10.1038/s41598-025-32614-6)
Supplement: Supplementary file 1 — Supplementary Information. [file 41598_2025_32614_MOESM1_ESM.docx]

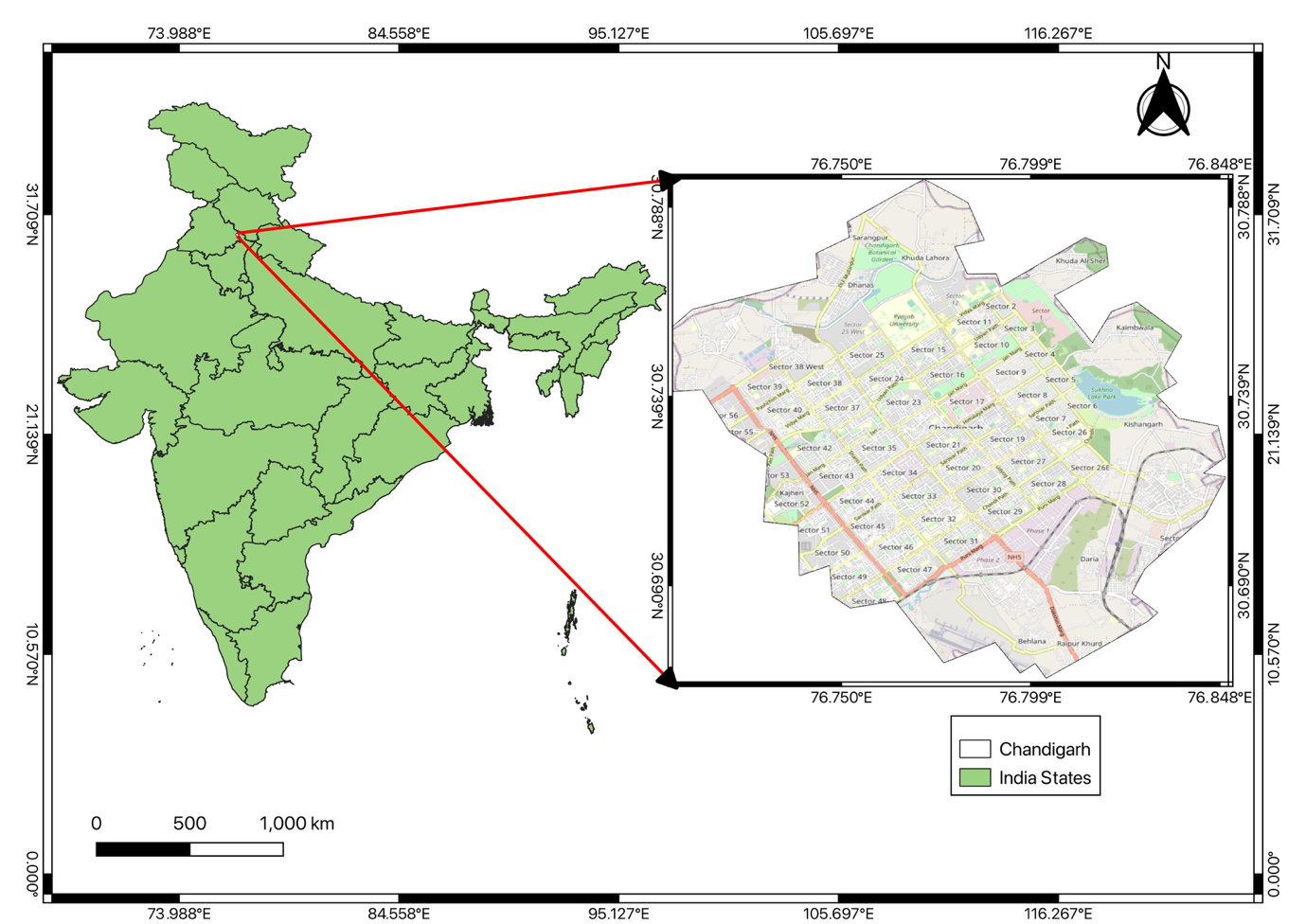


Figure S1: Map of Chandigarh City, India. Map created using QGIS [version 3.40] (https://www.qgis.org/) with administrative boundary data from Survey of India (https://onlinemaps.surveyofindia.gov.in/) and base layer from OpenStreetMap (https://www.openstreetmap.org/).


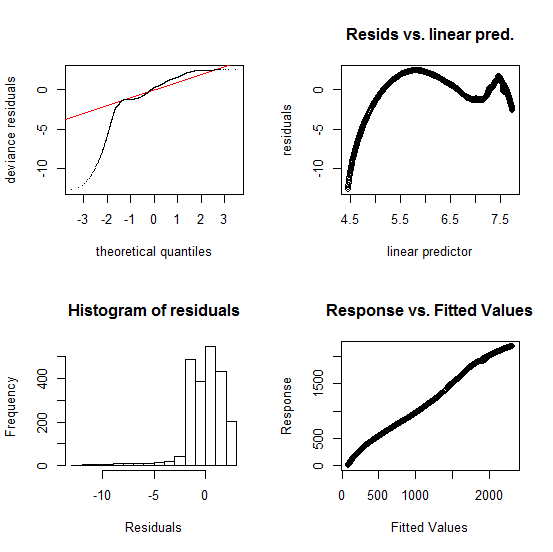


Figure S2: Summary statistics of the Generalized Additive Model (GAM) model applied to study the excess mortality associated with extreme temperature events in Chandigarh, India.


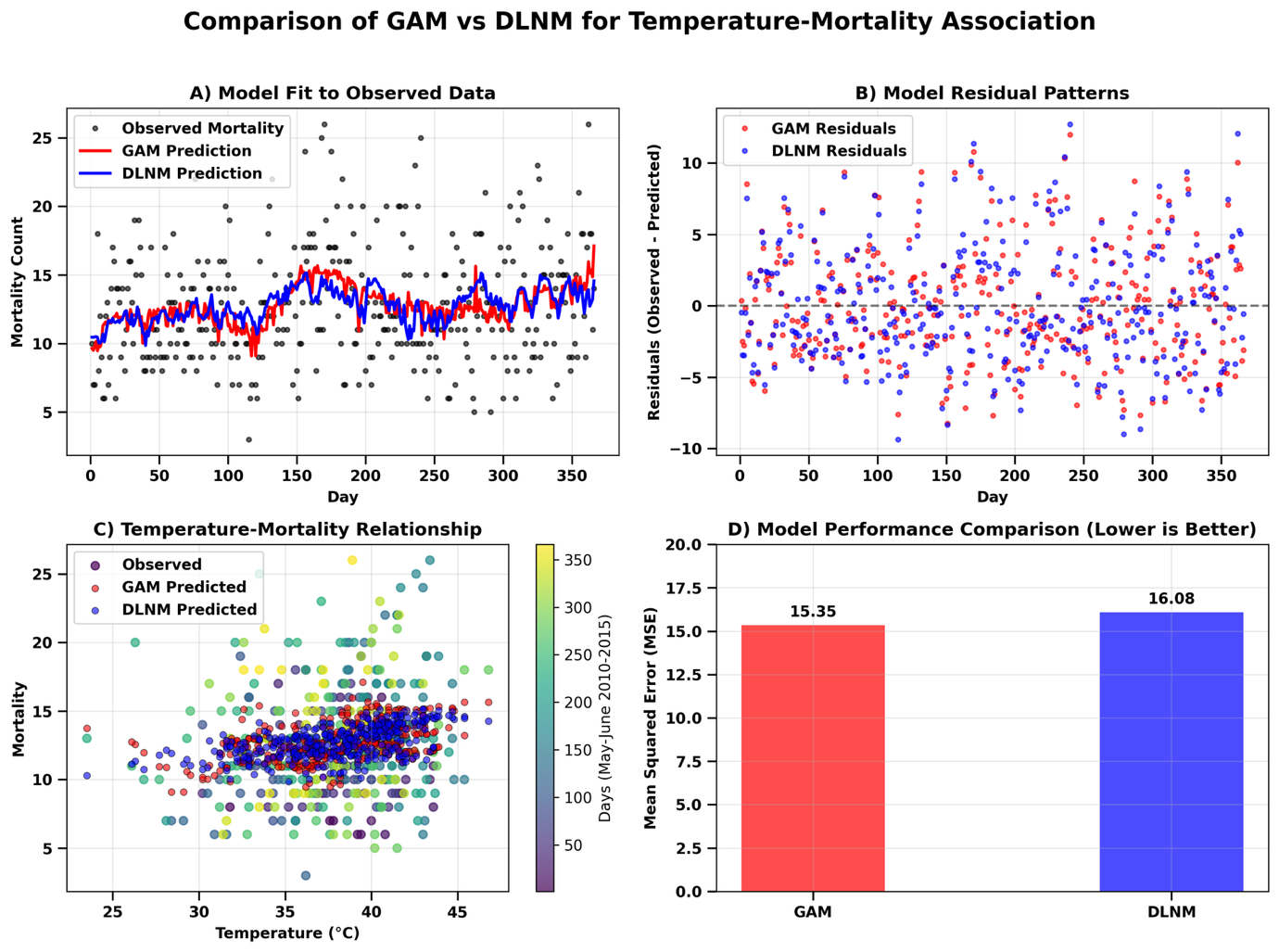


Figure S3: Comparison between the Standard over-dispersed Poisson Generalized Additive Model and a Distributed Lag Non-Linear Model (DLNM) (a) model fit to observed data (b) Model Residual Patterns (c) Temperature-Mortality Relationship (d) Model Performance Comparison


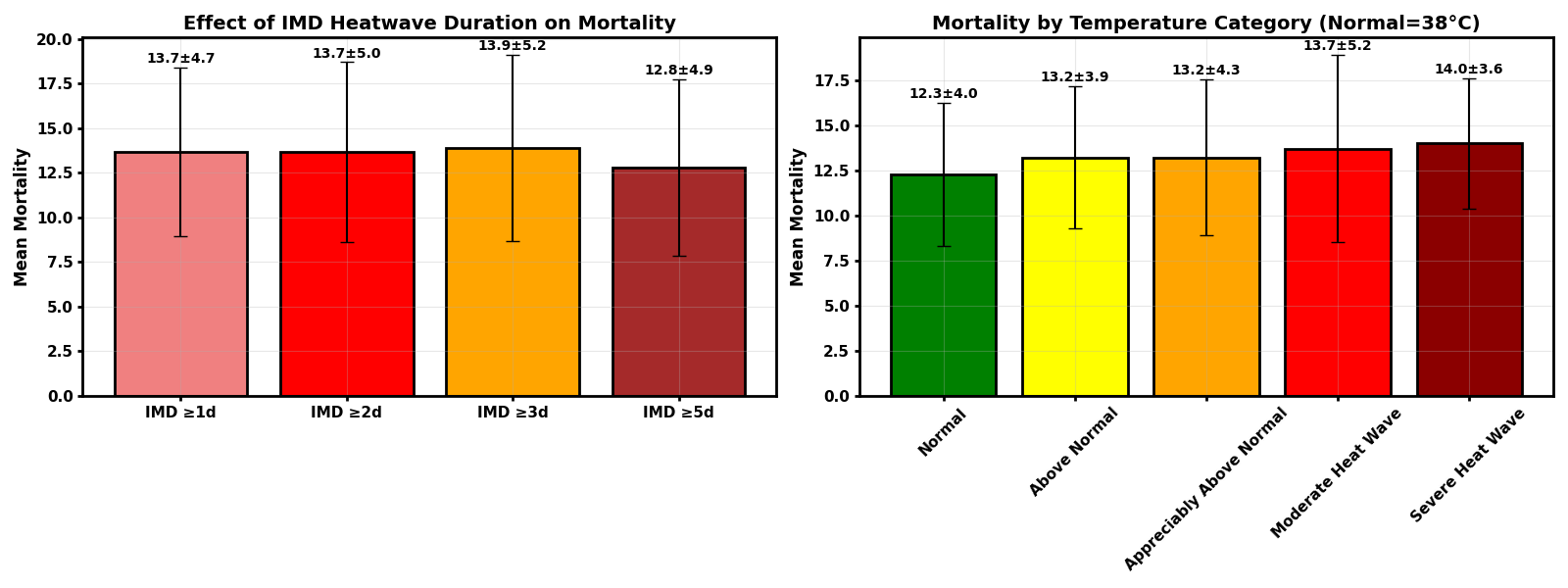


**Figure S4. The left panel explains mean mortality with a lagged effect, and the right panel explains mean mortality with different IMD criteria.**

**
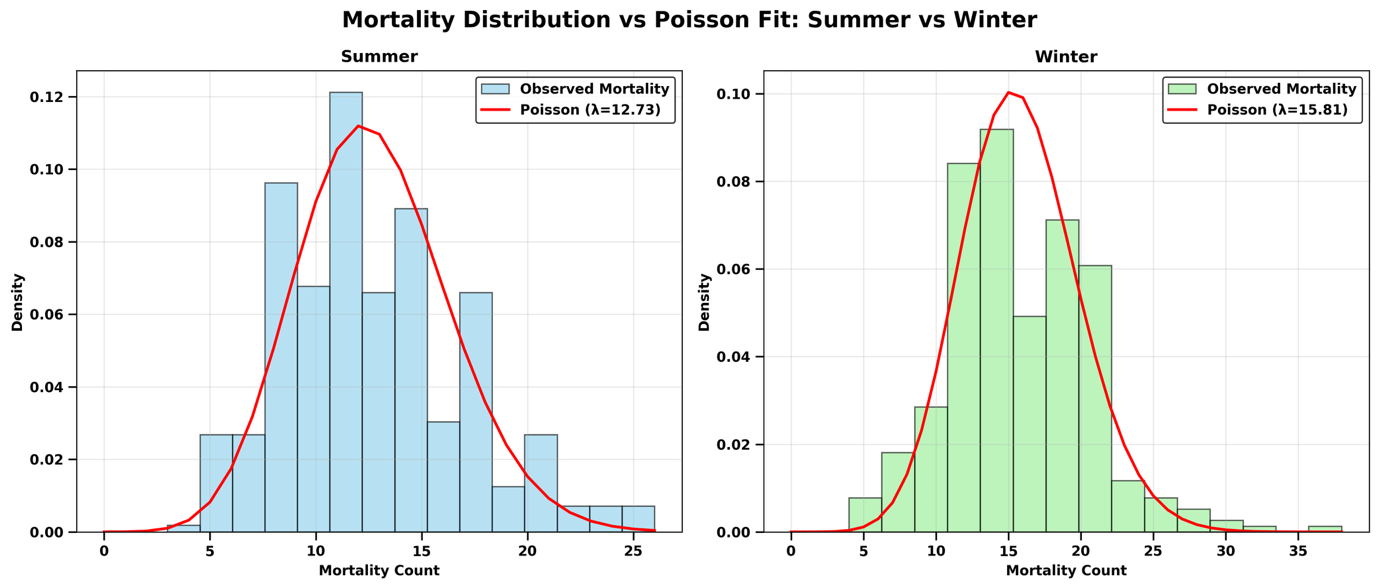
**

(a)

(b)

Figure S5: Mortality Distribution Vs Poisson Fit (a) in Summer (b) in Winters


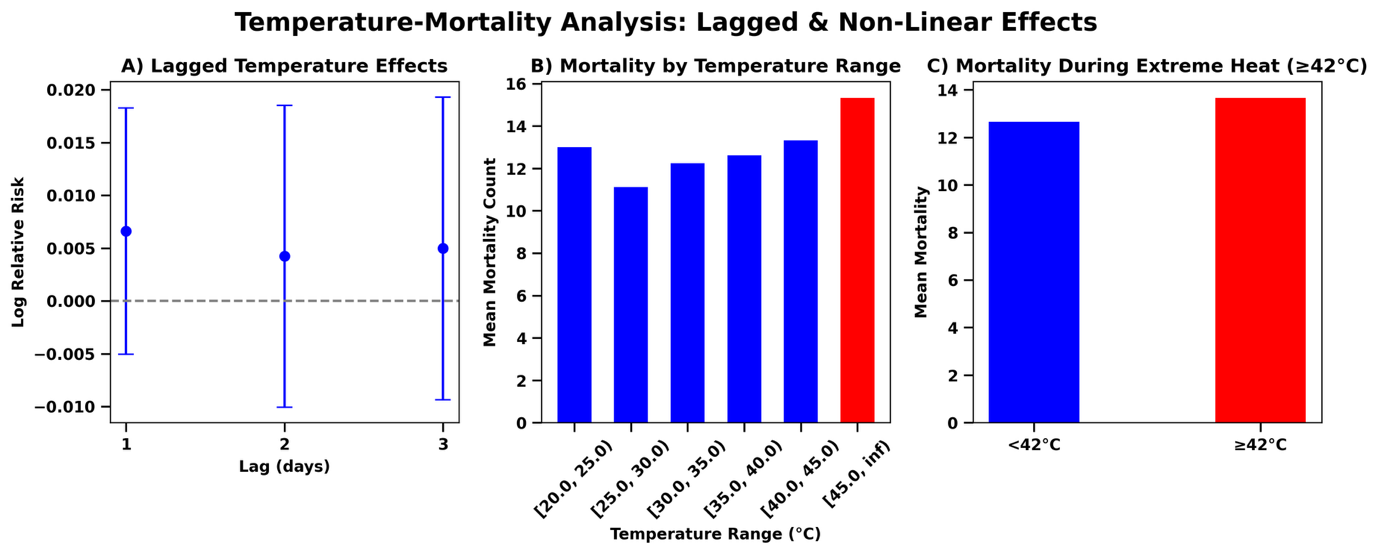


Figure S6: (a) the lagged temperature effect on mortality, (b) temperature range and associated mortality, and (c) mortality during extreme heat.

Table S1: Month-wise correlation between daily maximum temperature and daily all-cause mortality count

| **Year** | **May** | **June** | **December** | **January** |
| --- | --- | --- | --- | --- |
| **2010** | .067 | .2 | -0.04 | -.33 |
| **2011** | .34 | .55 | -.26 | -.27 |
| **2012** | .98 | .22 | -.21 | -.012 |
| **2013** | .145 | .36 | -.029 | -.136 |
| **2014** | .232 | .01 | -.023 | -.065 |
| **2015** | .039 | .20 | Few days data missing | -.80 |
